# Lack of collagen VI promotes neurodegeneration by impairing autophagy and inducing apoptosis during aging

**DOI:** 10.18632/aging.100924

**Published:** 2016-04-07

**Authors:** Matilde Cescon, Peiwen Chen, Silvia Castagnaro, Ilaria Gregorio, Paolo Bonaldo

**Affiliations:** ^1^ Department of Molecular Medicine, University of Padova, I-35131 Padova, Italy

**Keywords:** collagen VI, extracellular matrix, aging brain, apoptosis, autophagy

## Abstract

Collagen VI is an extracellular matrix (ECM) protein with a broad distribution in different tissues and mostly deposited at the close periphery of the cell surface. Previous studies revealed that collagen VI protects neurons from the toxicity of amyloid-βpeptides and from UV-induced damage. However, the physiological role of this protein in the central nervous system (CNS) remains unknown. Here, we established primary neural cultures from murine cortex and hippocampus, and carried out *in vitro* and *in vivo* studies in wild-type and collagen VI null (*Col6a1*^−/−^) mice. *Col6a1*^−/−^ neural cultures displayed an increased incidence of spontaneous apoptosis and higher vulnerability to oxidative stress, accompanied by altered regulation of autophagy with increased p62 protein levels and decreased LC3 lipidation. Analysis of brain sections confirmed increased apoptosis and abnormal regulation of autophagy in the CNS of collagen VI-deficient animals. To investigate the *in vivo* physiological consequences of these CNS defects, we carried out functional studies and found that motor and memory task performances were impaired in aged *Col6a1*^−/−^ mice. These findings indicate that lack of collagen VI leads to spontaneous apoptosis and defective autophagy in neural cells, and point at a protective role for this ECM protein in the CNS during physiological aging.

## INTRODUCTION

Collagen VI is an ECM protein with a broad distribution in several tissues including skin, tendons, lungs, skeletal muscles, heart, adipose tissue, cartilages and peripheral nerves [1]. Typically composed by three genetically distinct polypeptide chains, α1(VI), α2(VI) and α3(VI), collagen VI monomers undergo intra-cellular assembly into disulfide-bonded dimers and tetramers, which are finally secreted outside the cell, giving rise to the characteristic beaded microfilaments within the ECM [2, 3, 4].

In the CNS, collagen VI was first described among the ECM molecules detected in the proper glia limitans [5]. Initial immunohistochemical studies showed the presence of collagen VI mainly in the connective compartments of the CNS [6]. More recently, a study demonstrated increased hippocampal *Col6a1* expression in a transgenic mouse model for familial Alzheimer's disease, as well as in patients with Alzheimer's disease, and suggested a neuroprotective role for collagen VI against the toxicity of amyloid-Δ peptides [7]. A protective effect for collagen VI was also suggested by studies on UV irradiation in cultured neurons [8]. Although these data imply that collagen VI exerts a protective effect in the CNS under stress or damage conditions, the physiological role of collagen VI in the CNS remains unknown as no study was ever carried out in the absence of experimentally induced neuronal damage.

The availability of a collagen VI null mouse model, generated by targeted inactivation of the *Col6a1* gene, allowed shedding light into the role of collagen VI in specific tissues [9]. Mutations of collagen VI genes in humans are known to cause a range of inherited muscle diseases, including Bethlem myopathy, Ullrich congenital muscular dystrophy and myosclerosis [10, 11]. Therefore, most studies on collagen VI null (*Col6a1*^−/−^) mice were focused on skeletal muscle, leading to the elucidation of the pathophysiological defects affecting muscles, which include ultrastructural alterations of mitochondria and sarcoplasmic reticulum, latent mitochondrial dysfunction, increased ROS production and spontaneous apoptosis of myofibers [9, 12, 13]. Recently, we demonstrated that a failure of the autophagy-lysosome system in collagen VI deficient myo-fibers is responsible for the accumulation of damaged organelles, leading to functional muscle defects [14].

Apoptosis and autophagy are cellular processes of utmost importance for the homeostasis of the CNS, where defective regulation of the autophagic flux can lead to neurodegenerative features [15, 16]. To get insight into the role of collagen VI in the CNS compartment, we took advantage of the *Col6a1* knockout mouse model and performed *in vitro* and *in vivo* studies. We found that lack of collagen VI causes defective regulation of autophagy, increased susceptibility to oxidative stress and spontaneous apoptosis of neural cells, leading to functional CNS alterations in aged mice. These findings demonstrate that collagen VI exerts a critical role in the CNS under physiological conditions, pointing at this ECM molecule as a protective factor during aging.

## RESULTS

### Collagen VI is produced by primary neural cell cultures

**Figure 1:**
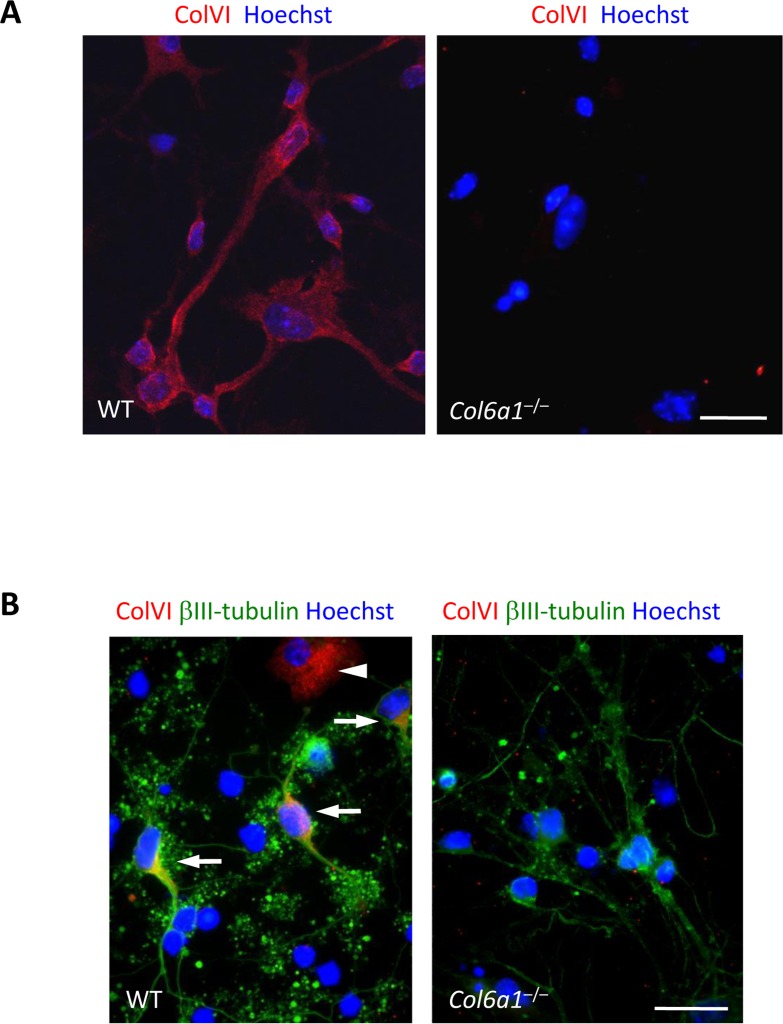
Collagen VI immunolabeling in primary neural cell cultures (**A**) Confocal microscopy analysis of immune-fluorescence for collagen VI in primary neural cell cultures. Collagen VI (red) is detected in wild-type, but not in *Col6a1*^−/−^ cultures. Nuclei were stained with Hoechst (blue). Scale bar, 100 μm. (**B**) Immunofluorescence for collagen VI (red) and βIII-tubulin (green). In wild-type cultures, collagen VI labelling is detected in both neuronal (βIII-tubulin-positive, arrows) and glial (βIII-tubulin-negative, arrowhead) cells. Nuclei were stained with Hoechst (blue). Scale bar, 100 μm. ColVI, collagen VI; WT, wild-type.

We established primary neural cell cultures from hippocampi and cortices of wild-type and *Col6a1*^−/−^ newborn (P0-P1) mice. Immunofluorescence showed lack of collagen VI labelling in *Col6a1*^−/−^ cultures, as expected (Fig. 1A). In wild-type cultures, collagen VI was detected in the proximity of the plasma membrane of cultured cells. No labelling for collagen VI was instead detectable in the interstitial spaces between cells, thus indicating that in these culture conditions the protein is in close contact with the cell surface and does not form an extensive fibrillar network. Double immunofluorescence showed that collagen VI labelling was present on both βIII-tubulin-positive and -negative cells (Fig. 1B).

### Lack of collagen VI causes spontaneous apoptosis in neural cell cultures

**Figure 2:**
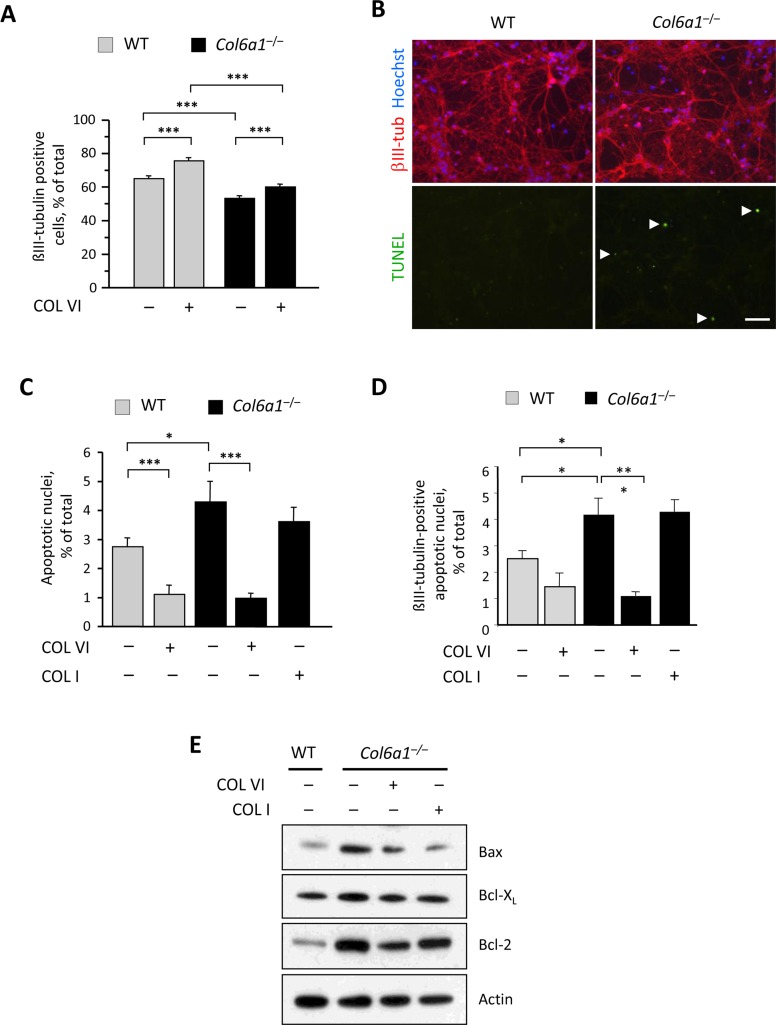
Lack of collagen VI affects the neuronal population and induces increased apoptosis in neural cell cultures (**A**) Quantification of the percentage of βIII-tubulin-positive cells in primary neural cell cultures derived from wild-type and *Col6a1*^−/−^ mice, grown in the absence (−) or in the presence (+) of purified collagen VI as a substrate (***, *P*<0.001; *n* = 6). (**B**) Representative images of TUNEL analysis in primary neural cell cultures derived from wild-type and *Col6a1*^−/−^ mice. Neurons were stained by immunofluorescence for βIII-tubulin (red, upper panels), and nuclei were labelled with Hoechst (blue, upper panels). TUNEL-positive nuclei are green (arrowheads, lower panels). Scale bar, 50 μm. (**C,D**) Quantification of TUNEL-positive nuclei in all cells (**C**) and of TUNEL-positive nuclei in βIII-tubulin-positive cells (**D**) in primary neural cell cultures derived from wild-type and *Col6a1*^−/−^ mice, grown in the absence (−) or in the presence (+) of collagen VI or collagen I as substrates (***, *P*<0.001; *, *P*<0.05; *n* = 3). (**E**) Western blot analysis of pro- and anti-apoptotic factors in total protein extracts derived from wild-type and *Col6a1*^−/−^ primary neural cell cultures. Actin was used as a loading control. COL I, purified collagen I; COL VI, purified collagen VI; WT, wild-type.

Previous studies indicated that collagen VI exerts a protective effect when neurons are treated with amyloid-β peptides or upon UV-irradiation [7, 8]. In our primary cell culture model, and in the absence of any specific stress challenge, wild-type cultures exhibited higher amount of neurons when compared to collagen VI null cultures (Fig. 2A), suggesting that collagen VI is beneficial for neurons even under physiological culture conditions. To further assess this, we cultured wild-type and *Col6a1*^−/−^ neural cells onto purified native collagen VI, provided as an adhesion substrate, and found that the number of neurons was significantly higher in cultures of both genotypes grown on purified collagen VI, when compared to the corresponding cultures grown on poly-lysine (Fig. 2A), suggesting that the protein can either support cell viability and/or favor cell adhesion.

Next, we investigated whether lack of collagen VI may affect the survival of neural cells. TUNEL analysis showed that *Col6a1*^−/−^ neural cultures had a significantly higher incidence of spontaneous apoptosis than that displayed by the corresponding wild-type cultures (Fig. 2 B and C). Notably, the incidence of TUNEL-positive nuclei was significantly lower in cultures of both genotypes when cells were seeded on collagen VI, but not when they were seeded on collagen I (Fig. 2C), thus indicating that collagen VI elicits an anti-apoptotic effect, that is not mimicked by another abundant ECM component, such as collagen I. Similar results were obtained when considering only the βIII-tubulin-positive cells in the cultures, suggesting that the neuronal fraction is the cell population mostly affected by lack of collagen VI (Fig. 2D). Western blot analysis for pro- and anti-apoptotic factors in protein extracts derived from wild-type and *Col6a1*^−/−^ neural cultures showed increased levels of Bax in *Col6a1*^−/−^ samples when compared to wild-type samples. Conversely, the anti-apoptotic factor Bcl-X_L_ displayed apparently similar levels in cultures of both genotypes, whereas Bcl-2 was noticeably increased in *Col6a1*^−/−^ cultures (Fig. 2E).

### Autophagic flux is deregulated in *Col6a1*^−/−^ neural cell cultures

**Figure 3:**
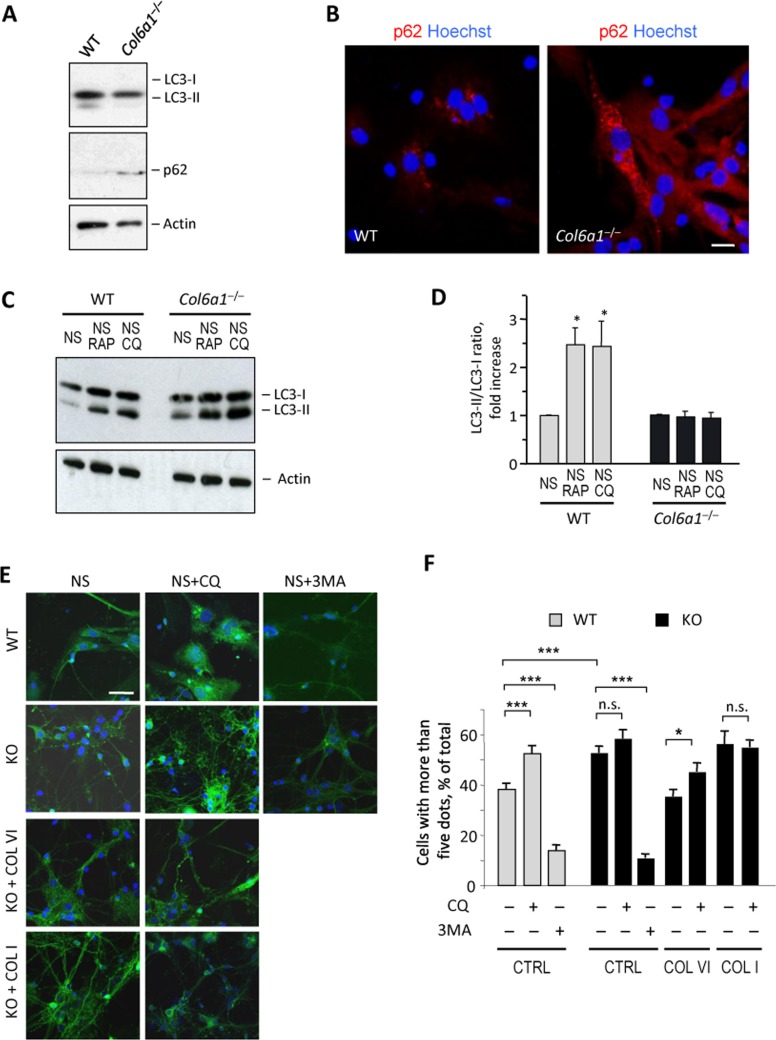
Col6a1−/− neural cell cultures display altered autophagic flux (**A**) Western blot analysis of LC3 and p62 in protein lysates from wild-type and *Col6a1*^−/−^ primary neural cell cultures. Actin was used as a loading control. (**B**) Immunofluorescence for p62 (red) in wild-type and *Col6a1*^−/−^ primary neural cultures, showing accumulation of p62 aggregates in *Col6a1*^−/−^ cells. Nuclei were stained with Hoechst (blue). Scale bar, 100 μm. (**C**) Western blot analysis for LC3 in protein lysates from wild-type and *Col6a1*^−/−^ primary neural cultures maintained for 4.5 hr in serum-free medium (NS), serum-free medium with 100 nM rapamycin (NS RAP), or serum-free medium with 50 μM chloroquine (NS CQ). Actin was used as a loading control. (**D**) Densitometric quantification of the LC3-II/LC3-I ratio, as determined by western blot analysis of three independent primary neural cultures from wild-type and *Col6a1*^−/−^ mice. The LC3-II/LC3-I ratio is expressed as fold-change relative to the serum-free condition (*, *P*<0.05; *n* = 3). (**E**) Fluorescence microscopy analysis of GFP-LC3 distribution in neural cell cultures derived from GFP-LC3::*Col6a1^+/+^* (WT) and GFP-LC3::*Col6a1*^−/−^ (KO) mice and maintained for 4.5 hr in serum-free medium in the absence (NS) or in the presence of 50 μM chloroquine (NS+CQ) or of 10 mM 3-methyladenine (NS+3MA). Where indicated, before the experiment cells were seeded onto purified collagen VI (COL VI) or collagen I (COL I) as substrates. Nuclei were stained with Hoechst (blue). Scale bar, 50 μm. (**F**) Quantification of GFP-LC3 puncta in neural cell cultures derived from GFP-LC3::*Col6a1^+/+^* (WT) and GFP-LC3::*Col6a1*^−/−^ (KO) mice and maintained in the different conditions described above. The histogram shows the percentage of cells with more than five fluorescent puncta for each condition (***, *P*<0.001; *, *P*<0.05; n.s., not significant; *n* = 3). 3MA, 3-methyl-adenine; COL I, adhesion onto collagen I; COL VI, adhesion onto collagen VI; CQ, chloroquine; CTRL, adhesion onto poly-lysine; WT, wild-type.

We previously demonstrated that lack of collagen VI leads to inefficient regulation of autophagy in muscle fibers, with impaired autophagic flux [14]. Considering the importance of a proper autophagic activity for CNS homeostasis, we investigated autophagy in wild-type and *Col6a1*^−/−^ neural cell cultures. Western blot analysis of microtubule-associated protein-1 light chain 3 (LC3), a well-established autophagic marker that undergoes lipidation during autophagosome formation [17, 18], showed decreased levels of lipidated LC3 in *Col6a1*^−/−^ neural cultures (Fig. 3A). In addition, *Col6a1*^−/−^ neural cultures displayed higher protein levels for p62 (Fig. 3A), a substrate of the autophagy-lysosome system that inversely correlates with autophagic activity [18, 19]. The higher p62 levels were matched by the markedly increased presence of p62 aggregates in *Col6a1*^−/−^ cultures (Fig. 3B). These results suggested defective regulation of autophagy in *Col6a1*^−/−^ neural cultures. To investigate further this aspect, we evaluated the ability of wild-type and *Col6a1*^−/−^ neural cultures to activate autophagy following different stimuli. Towards this aim, we maintained cells in serum-free medium in the absence or presence of rapamycin, a well-known autophagy inducer [20], and chloroquine, a lysosomal inhibitor that blocks the fusion and degradation of autophagosomes [21]. As expected, wild-type cultures displayed increased LC3 lipidation when either rapamycin or chloroquine was added to serum-free medium. Conversely, neither rapamycin nor chloroquine was able to elicit any significant increase of LC3 lipidation in serum-free *Col6a1*^−/−^ cultures (Fig. 3 C and D), thus confirming defective regulation of autophagy in collagen VI null neural cells.

To monitor the autophagic flux in more detail, we derived primary neural cell cultures from wild-type and *Col6a1*^−/−^ mice that were previously crossed with transgenic animals ubiquitously expressing GFP-tagged LC3 [22], thus allowing to monitor LC3-I to LC3-II conversion by following the redistribution of the fluorescent protein from a diffuse cytosolic to a punctuate localization due to its recruitment on autophagosome membranes. Primary neural cells derived from GFP-LC3::*Col6a1*^+/+^ mice and grown in serum-free medium for 4.5 hr displayed a punctuate LC3 fluorescence, consistent with autophagosome formation (Fig. 3 E and F). As expected, following the addition of chloroquine, GFP-LC3::*Col6a1*^+/+^ cells displayed an increased number of fluorescent puncta, whereas they displayed a dramatic decrease when treated with 3-methyladenine (3MA), a class III PI3-kinase inhibitor that inhibits the lipid kinase complex required for the formation of the autophagosome isolation membrane [23] (Fig. 3 E and F). GFP-LC3::*Col6a1*^−/−^ cells displayed a higher number of fluorescent puncta than wild-type cells after culture in serum-free medium for 4.5 hr. Moreover, treatment with chloroquine did not achieve any significant increase in the amount of fluorescent autophagosomes in GFP-LC3::*Col6a1*^−/−^ cells (Fig. 3 E and F), whereas a clear response to 3MA was detected in these cells, with a marked decrease of fluorescent puncta, thus indicating that they were able to modulate auto-phagosome formation. Adhesion of LC3::*Col6a1*^−/−^ cultures onto purified native collagen VI led to a decreased number of fluorescent autophagosomes, with similar levels to those displayed by wild-type cells, and restored the response to chloroquine treatment (Figure 3 E and F). Interestingly, adhesion of GFP-LC3::*Col6a1*^−/−^ cultures onto a different ECM substrate, collagen I, did not elicit any significant response on the number of fluorescent autophagosomes.

### *Col6a1*^−/−^ neural cell cultures are more vulnerable to oxidative damage

**Figure 4:**
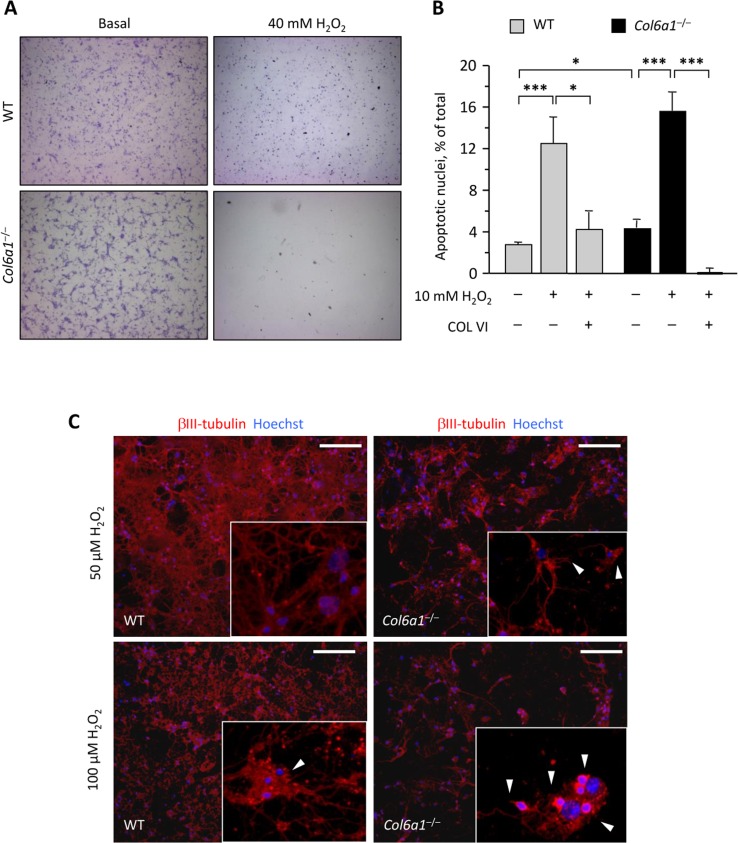
Col6a1−/− neural cell cultures display higher oxidative damage (**A**) Light microscopy analysis of wild-type and *Col6a1*^−/−^ primary neural cell cultures maintained in standard condition (Basal) or after treatment for 90 min with 40 mM H_2_O_2_. Cells were fixed and stained with cresyl violet. Scale bar, 100 μm. (**B**) Quantification of TUNEL-positive nuclei in wild-type and *Col6a1*^−/−^ primary neural cell cultures maintained in standard condition or after treatment for 90 min with 10 mM H_2_O_2_. Where indicated, cells were grown onto purified collagen VI before treatment (***, P < 0.001; *, *P*<0.05; *n* = 3). COL VI, adhesion onto purified collagen VI. WT, wild-type. (**C**) Immunofluorescence for βIII-tubulin (red) in wild-type and *Col6a1*^−/−^ neural cell cultures after treatment for 90 min with 50 μM H_2_O_2_ (top panels) or 100 μM H_2_O_2_ (bottom panels). Even at lower doses of hydrogen peroxide, *Col6a1*^−/−^ cultures display a less dense neuronal network, with higher incidence of dendrite shrinkage (arrowheads in insets). The insets show higher magnification details of each panel. Nuclei were stained with Hoechst (blue). Scale bar, 50 μm. WT, wild-type.

Considering that autophagy impairment may lead to several defects within the cells such as ROS accumulation, rendering them prone to oxidative damage [24], we induced oxidative stress in wild-type and *Col6a1*^−/−^ primary neural cells by treating cultures with different doses of hydrogen peroxide. Treatment with 40 mM H_2_O_2_ for 90 min induced a dramatic cell loss in *Col6a1*^−/−^ cultures, at a much higher extent to that observed in wild-type cells (Fig. 4A). Interestingly, adhesion onto collagen VI as a substrate led to a higher resistance to milder (10 mM) H_2_O_2_ treatments in cultures of both genotypes (Fig. 4B). In order to further evaluate differences in the sensitivity to oxidative damage between the two genotypes, we exposed neural cell cultures to micromolar concentrations of hydrogen peroxide. Interestingly, at both 50 μM H_2_O_2_ and 100 μM H_2_O_2_, *Col6a1*^−/−^ cells showed several signs of degeneration, such as loss of neuronal network and dendrite shrinkage, whereas wild-type cells appeared much healthier at both H_2_O_2_ concentrations (Fig. 4C).

### Aged *Col6a1*^−/−^ mice display hallmarks of neurodegeneration

**Figure 5:**
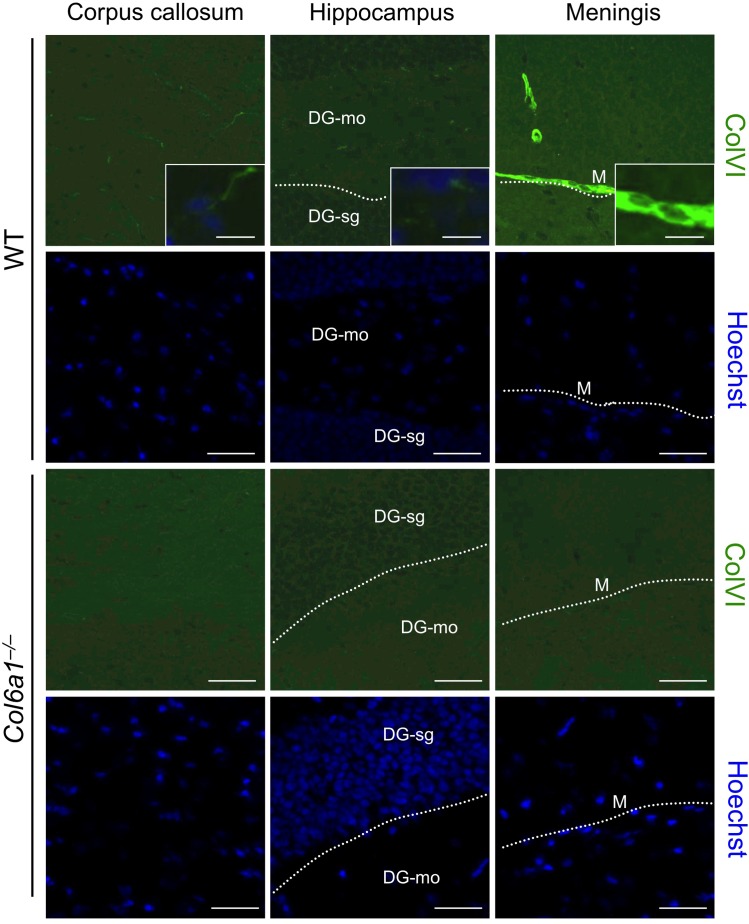
Collagen VI detection in adult mouse brain Immunofluorescence for collagen VI on brain sections of wild-type and *Col6a1*^−/−^ adult mice. Labelling for collagen VI is detectable in different regions, in particular in corpus callosum, within the hippocampus and along meninges. Nuclei were stained with Hoechst (blue). Scale bar in panels, 40 μm; scale bar in insets, 15 μm. DG-mo, dentate gyrus, molecular layer; DG-sg, dentate gyrus, granule cell layer; M, meningis; WT, wild-type.

**Figure 6:**
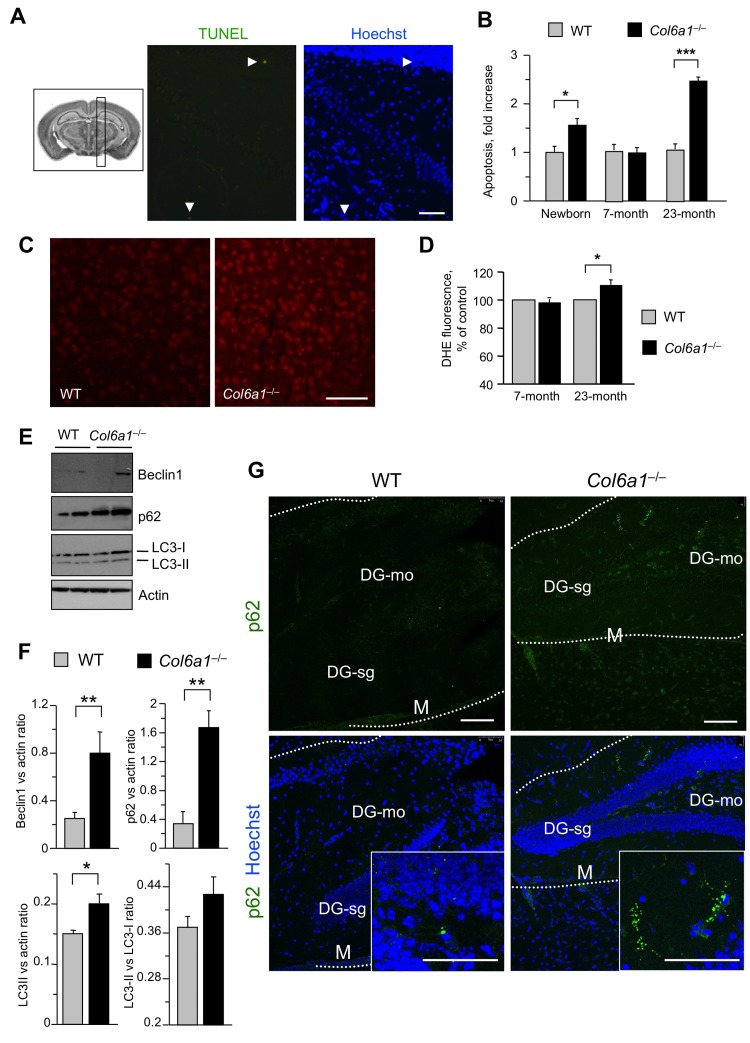
Aged Col6a1−/− mice display neurodegenerative hallmarks (**A**) Representative image of TUNEL assay in adult *Col6a1*^−/−^ mouse brain sections derived from the region showed in the inset on the left. TUNEL-positive nuclei (green) are shown in the middle panel. Nuclei were stained with Hoechst (blue, right panel). Scale bar, 50 μm. (**B**) Quantification of TUNEL-positive nuclei in sagittal sections of brains of newborn, 7-month-old and 23-month-old wild-type and *Col6a1*^−/−^ mice. The number of TUNEL-positive cells was counted per area unit (mm^2^), and the incidence of apoptosis in *Col6a1*^−/−^ mice calculated as fold-change relative to the age-matched wild-type values (***, *P*<0.01; *, *P*<0.05; *n* = 3-7). (**C**) Representative images of DHE staining in brain sections of 23-month-old wild- type and *Col6a1*^−/−^ mice. Scale bar, 100 μm. (**D**) Quantification of DHE fluorescence in brain sections of 7-month-old and 23-month-old wild-type and *Col6a1*^−/−^ mice. Values for *Col6a1*^−/−^ mice are reported as percentage relative to the age-matched wild-type value. ROS accumulation is significantly higher in aged *Col6a1*^−/−^ mice (*, *P*<0.05; *n* = 3-7). (**E**) Western blot analysis of the autophagic markers Beclin 1, p62 and LC3 in total protein extracts from brain of 23-month-old wild-type and *Col6a1*^−/−^ mice. Actin was used as a loading control. (**F**) Densitometric quantification of Beclin 1/actin ratio, p62/actin ratio, LC3-I/actin ratio and LC3-II/LC3-I ratio, as determined by three independent western blot experiments of brain extracts from 23-month-old wild-type and *Col6a1*^−/−^ mice. (**, P < 0.01; *, *P*<0.05; *n* = 3). (**G**) Immunofluorescence for p62 in brain sections from 23-month-old wild-type and *Col6a1*^−/−^ mice, revealing increased p62 labeling (green) in *Col6a1*^−/−^ samples. Insets show p62 aggregates at higher magnifications. Scale bar, 100 μm. DG-mo, dentate gyrus, molecular layer; DG-sg, dentate gyrus, granule cell layer; M, meningis; WT, wild-type.

To assess whether the alterations detected in *Col6a1*^−/−^ neural cells reflected phenotypic CNS defects in collagen VI-deficient mice, we carried out different *in vivo* studies. First, we performed immunofluorescence on brain sections of adult mouse in order to determine the localization of collagen VI in the regions from which neural cultures were derived in pups. Immunofluorescence for collagen VI protein showed labelling along meninges and blood vessels (Suppl. Fig.1), in agreement with previous studies on collagen VI distribution [5, 25]. In addition, higher magnifications revealed that the protein is also present in the hippocampal region, as well as in corpus callosum (Fig. 5). In order to understand the *in vivo* significance of the cytoprotective role displayed by collagen VI in primary neural cultures, we performed studies aimed at investigating the presence of possible neurodegenerative hallmarks in brain sections from newborn (2-day-old), adult (7-month-old), and aged (23-month-old) *Col6a1^−/−^* mice. TUNEL analysis revealed a significantly increased number of apoptotic nuclei in brain samples of newborn *Col6a1^−/−^* mice when compared to the corresponding wild-type samples. Notably, although adult brains of the two genotypes displayed similar numbers of apoptotic nuclei, the incidence of TUNEL-positive nuclei was significantly higher in brain sections from 23-month-old *Col6a1^−/−^* mice (Fig. 6 A and B), whereas the number of total neurons does not appear significantly altered in *Col6a1*^−/−^ aged brains (Suppl. Fig. 2). Interestingly, real time RT-PCR and immunofluorescence analysis of wild-type samples showed that expression and deposition of collagen VI is increased in aged mouse brains (Suppl. Fig. 3), prompting us to perform further *in vivo* studies on aged mice. In agreement with the *in vitro* data obtained on neural cultures, western blot of protein extracts from aged mouse brains revealed increased levels of Bcl-2 in *Col6a1^−/−^* samples when compared to age-matched wild-type samples (Suppl. Fig. 4). Since collagen VI null cultures also displayed higher sensitivity to oxidative stress, we evaluated ROS production in the brain of wild-type and *Col6a1^−/−^* mice by dihydroethidium (DHE) staining. Quantification of DHE fluorescence showed that ROS production was significantly higher in the brain of aged *Col6a1^−/−^* mice, when compared to age-matched wild-type samples, whereas younger mouse brains did not reveal any significant difference between the two genotypes (Fig. 6 C and D). These findings point at a protective role for collagen VI against age-induced oxidative damage.

Aging is a process tightly related to the decreased efficiency of the autophagic pathway, particularly in post-mitotic tissues such as CNS [26]. Because of this, and given the altered autophagic response detected in *Col6a1^−/−^* neural cultures, we analyzed some autophagic markers in protein extracts of aged wild-type and *Col6a1^−/−^* mouse brains. Extracts from aged *Col6a1^−/−^* brains displayed increased levels of Beclin 1 (Fig. 6 E and F), a key regulator of autophagosome formation, pointing at a stronger induction of autophagy initiation. Interestingly, p62 levels and LC3-II/actin ratio were also significantly increased in aged *Col6a1^−/−^* brains (Fig. 6 E and F). Immunofluorescence analysis of brain sections from aged mice confirmed that p62 was present at higher levels in aged *Col6a1^−/−^* brains and also revealed the presence of p62 aggregates (Fig. 6G), partially co-localizing with ubiquitin staining (Suppl. Fig. 5). These findings indicate that the autophagic pathway is dysregulated in the CNS of aged *Col6a1*^−/−^ mice.

**Figure 7:**
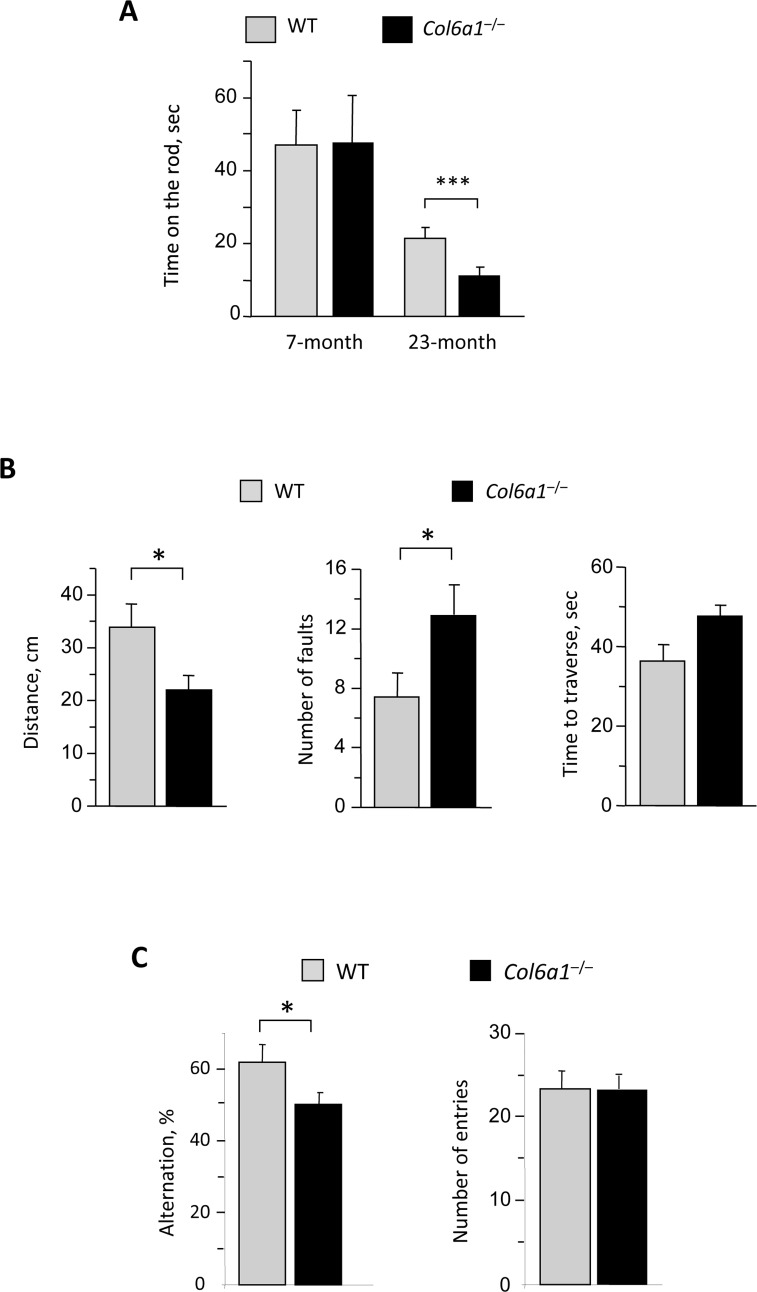
Reduced motor and memory tasks in aged Col6a1−/− mice (**A**) Rotarod test analysis on 7-month-old and 23-month-old wild-type and *Col6a1*^−/−^ mice. The time on the rod (in sec) is reported in the graph. Aged *Col6a1*^−/−^ animals show reduced time to fall from rotarod compared to matched wild-type animals (***, *P*<0.001; *n* = 4). (**B**) Ledged beam test results from 23-month-old wild-type and *Col6a1*^−/−^ mice. The histograms report the mean distance before the initial foot fault, the number of total hindpaw foot faults, and the time spent to reach the end of the beam (*, *P*<0.05; *n* = 4). (**C**) Y-maze test results from 23-month-old wild-type and *Col6a1*^−/−^ mice. The histograms report the percentage of alternation and the number of entries (*, *P*<0.05; *n* = 8-15).

Impaired motor coordination was previously reported as a result of defective autophagy in mice with neural cell-specific ablation of Atg5 and Atg7 [15, 16]. In agreement with this, rotarod performance was significantly reduced in aged *Col6a1*^−/−^ mice when compared to age-matched wild-type mice, whereas younger animals of the two genotypes displayed similar rotarod performance (Fig. 7A). Moreover, fine motor coordination and balance were impaired in aged *Col6a1*^−/−^ mice as revealed by ledged beam test (Fig. 7B). The first foot fault occurred significantly earlier in aged *Col6a1*^−/−^ mice and the total number of faults was higher than in age-matched wild-type controls (Fig. 7B). Interestingly, not only motor coordination but also spatial memory task was significantly reduced in aged *Col6a1*^−/−^ mice, as revealed by Y-maze test, indicating a reduced alternation between different entries, whereas no difference was found in the total number of entries (Fig. 7C).

## DISCUSSION

Collagen VI is an ECM protein found in a wide range of tissues [25, 27, 28, 29] and exerting a cytoprotective role by counteracting apoptosis and oxidative damage in different cell types [12, 13, 30]. Although, previous works pointed at collagen VI as being protective for neurons against injury-induced cell death [7, 8], our studies in *Col6a1*^−/−^ mice reveal a key functional role for this protein within the CNS under physiological conditions. ECM collagens are absolutely uncommon in the mature CNS except for few types expressed by neurons, such as collagens XVI [31], XVII [32], XIII [33] and XXV [34], which are considered to fulfil functional, rather than merely structural roles in the CNS [35]. Although collagen VI expression in the CNS remains to be characterized in further detail, our data indicate that collagen VI deposition is not only limited to the CNS-related connective tissues, but also extends to proper neural tissues. Moreover, our *in vitro* and *in vivo* data demonstrate that collagen VI exerts its protective role in the CNS by influencing apoptosis and autophagy, two key intracellular processes of utmost importance for CNS homeostasis and with relevant roles in neurodegenerative diseases [36].

An impaired autophagic activity has been shown to be sufficient to induce marked neurodegenerative hallmarks in mice [15, 16]. Indeed our *in vitro* studies show that the sole serum withdrawal led to higher LC3 lipidation in *Col6a1*^−/−^ neural cultures, as revealed by western blot and by fluorescence microscope analysis of GFP-LC3 fluorescent puncta, without any significant change in response to the autophagy inducer rapamycin and the lysosomal inhibitor chloroquine, thus underlining the presence of a defect of the autophagy-lysosome system in neural cells lacking collagen VI. These data further suggest that serum depletion stresses so much *Col6a1*^−/−^ neural cells that they maximally induce autophagy, reaching a condition of exhaustion, without being able to complete the clearance of autophagosomes. Similar findings were reported for other *in vitro* and *in vivo* disease models, such as Vici syndrome and some neurodegenerative disorders, showing that defective autophagosomal clearance is accountable for increased evidence of autophagosomes even in the absence of lysosomal inhibitors or autophagy inducers [37, 38, 39]. Autophagy regulation in neurons is tightly connected with ROS production, and recent studies also pointed at an essential role for ROS in starvation-induced autophagy [40]. Conversely, several studies demonstrated that compromised autophagy or defective lysosomal activity can increase the presence of ROS in cultures as well as in mouse models for neurodegenerative diseases [24]. Notably, not only highly stressing doses [41] but also micromolar concentrations of hydrogen peroxide caused marked degenerative features in *Col6a1*^−/−^ neural cultures. All these findings underline that collagen VI is cytoprotective for neurons and that lack of this ECM component impinges on apoptosis, autophagy and oxidative damage in neural cell cultures.

Remarkably, similar defects were revealed by our *in vivo* studies in brain sections of aged *Col6a1*^−/−^ mice, thus indicating that ablation of collagen VI is detrimental for the CNS. In particular, apoptosis and ROS levels were markedly increased in brains of aged *Col6a1*^−/−^ mice, thus revealing that collagen VI exerts an *in vivo* cytoprotective role in the CNS and preserves the nervous tissue from oxidative damage, which is known to increase during aging [24]. Autophagic markers were also altered in brain sections of aged *Col6a1*^−/−^ mice, with a marked increase of p62 that also localized in aggregates partially positive for ubiquitin. The presence of p62 aggregates was previously reported in aging conditions, and p62 was shown to be a common component of neuronal cytoplasmic inclusions often found in patients affected by motor neuron diseases [42, 43]. In addition, Beclin 1 protein levels and LC3-II/actin ratio were markedly increased in brain sections of aged *Col6a1*^−/−^ mice when compared to age-matched wild-type samples, thus indicating that ablation of collagen VI in the CNS leads to a dysregulation of the autophagic flux during aging.

The autophagic defects displayed by *Col6a1*^−/−^ neural cell cultures and aged brains are particularly interesting. A wealth of studies in different tissues in both physiological and pathological conditions indicates that autophagy is essential for cell and tissue homeostasis, so that both inefficient or excessive induction of autophagy are detrimental [44, 45]. An increasing number of studies on aging models showing alterations in the autophagic pathway were reported. The senescence-accelerated mouse prone 8 (SAMP8), characterized by early manifestations of cognitive dysfunction and neurodegeneration [46, 47], displays alterations of autophagy, including increased LC3-II/actin ratio and a peculiar trend for Beclin 1 [48]. Alterations of Beclin 1 were reported in different neurodegenerative conditions: on the one hand, it is reduced in the brain of Alzheimer's disease patients, supporting enhanced neurodegeneration [49]; on the other hand, Beclin 1 was shown to be increased in some neurodegenerative disorders, where autophagy upregulation is needed for counteracting pathogenic proteins or injury [50]. Based on the findings we obtained in *Col6a1*^−/−^ mice, we can argue that a similar upregulated autophagic response is elicited by p62 aggregation, resulting in increased levels of Beclin 1 as a protective response, while the downstream efficiency of autophagy is already defective since both LC3-II/actin ratio and p62 levels are augmented. The complex network of effects involving autophagy in aging is also illustrated by the *Zmpste24* deficient mouse, a model for human Hutchinson-Gilford progeria, a type of accelerated aging that exhibits autophagy induction, rather than reduction, in connection with metabolic changes, such as lower insulin and glucose levels in blood, thus rising a novel paradoxical role for autophagy during pathological aging processes [51, 52].

In the onset of the neurodegenerative hallmarks of *Col6a1*^−/−^ mice, we cannot exclude a possible involvement of the blood-brain barrier (BBB), since collagen VI is abundantly deposited at the level of meninges. Although the role of collagen VI in the neurovascular unit was not investigated yet, several reports demonstrated that BBB leakage can impair brain microcirculation and cause the accumulation of blood-derived toxic proteins and agents, ultimately leading to neuronal loss [53]. Moreover, alterations in blood-spinal cord and blood-brain barriers were suggested to be involved in the onset of neuron degeneration in rodent models for amyotrophic lateral sclerosis [54] and in Alzheimer's disease patients, respectively [55]. In the latter case, BBB alterations were also linked to aging-induced minimal cognitive impairment.

Considering the surprisingly large number of interactions that collagen VI is capable to establish with different extracellular and cell surface molecules [4], addressing the nature of the specific receptors mediating collagen VI pro-survival effects in neural cells is a challenging task that should be accomplished in the next future. Some potential candidates include integrins α1β1, α2β1, α3β1, α10β1 and αvβ3, as well as the NG2/CSPG4 chondroitin sulphate proteoglycan [4]. Of note, NG2 is abundantly expressed within the nervous system, where it is a valuable marker for oligodendrocyte precursors populating both the grey and white matter [56] and for brain pericytes [57]. Studies on the localization of integrin subunits within brain regions reported the expression of α1, α2, α3, αv, β1 and β3 integrin chains in the cortex and hippocampus [58], where integrins were shown to be involved in dendritic spine remodeling [59], interneuron migrating trajectories [60] and in glial-mediated CNS repair [61]. Interestingly, some of the pathomolecular alterations found in the CNS are paralleled by similar defects in skeletal muscles of collagen VI deficient mice [12,13,14]. This suggests that transduction of collagen VI pro-survival signals may involve similar receptors in the muscle and in the brain. In this context, it is also worthy to consider that several muscle diseases have implications or correlations with CNS defects. The most known and studied example is provided by Duchenne muscular dystrophy [62], but CNS implications are also found in myotonic dystrophy [63]. Although no cognitive impairment was ever reported so far for patients affected by skeletal muscle diseases linked to collagen VI genes, such as Ullrich congenital muscular dystrophy and Bethlem myopathy [10], the evidence obtained in *Col6a1*^−/−^ mice supports the interest in deepening the studies on the occurrence of CNS alterations related to collagen VI defects. Along this line, it is interesting to consider that a recent study reported that a mutation of human *COL6A2* gene, coding for the α2 chain of collagen VI, is responsible for progressive myoclonus epilepsy syndrome [64]. In addition, mutations in the *COL6A3* gene were found in patients affected by recessive isolated dystonia, a human brain disorder [65].

In conclusion, our work highlights a protective role for collagen VI in the CNS during physiological aging. Our *in vitro* and *in vivo* data indicate that lack of collagen VI impinges on cell survival pathways, with increased apoptosis, oxidative damage and defective autophagy regulation, paralleled by motor and memory task impairment in aged mice, pointing at collagen VI as an ECM molecule exerting a distinct role in protecting CNS from aging.

## METHODS

### Mice

*Col6a1*^+/+^ (wild-type) and *Col6a1*^−/−^ mice in the C57BL/6 background [9, 12] were used in this study. 2-day, 7-month, and 23-month-old mice were sacrificed by cervical dislocation, brains were dissected and rapidly frozen in liquid nitrogen for protein extracts and histological analysis. Native collagen VI protein was purified from newborn mice as previously described [9, 12]. GFP-LC3 mice [22] were provided by Riken BRC (GFP-LC3#53 strain, RBRC00806). GFP-LC3::*Col6a1*^+/+^ and GFP-LC3::*Col6a1*^−/−^ mice were generated crossing *Col6a1*^−/−^ mice with GFP-LC3 animals. Mouse procedures were approved by the Ethics Committee of the University of Padova and authorized by the Italian Ministry of Health.

### Primary neural cultures

Cortices and hippocampi from newborn (P0-P1) wild-type and *Col6a1*^−/−^ mice were dissociated in trypsin (0.8 mg/ml, Sigma) for 10 min at 37°C. Digestion was blocked by a solution containing trypsin inhibitor (6.3 μg/ml, Sigma) and DNase I (40 μg/ml, Invitrogen). Dissociated cells were plated at a density of 2.5×10^5^ cells per cm^2^ well on glass coverslips or on plastic coated with either poly-L-lysine (100 μg/ml), collagen I (Sigma), or purified native murine collagen VI [12]. The culture medium consisted of MEM (GIBCO) containing glucose (20 mM), L-glutamine (0.5 mM), N2 supplement (1%), B27 supplement (0.5%), biotin (0.875 mg/l), pyruvic acid (1 mM), penicillin (25 μg/ml) streptomycin (25 μg/ml), fungizone (50 μg/ml) and horse serum (10%, Gibco). Cytosine-β-d-arabinofuranoside (3 μM) was added 24 hr after plating. Cultures were grown for 7 days, and the last day cells were subjected to different treatment conditions for 4.5 hr. The following treatments were used: DMEM (Gibco) without serum; DMEM without serum, in the presence of 3-methyladenine (10 mM, Sigma); DMEM without serum, in the presence of chloroquine (50 μM, Sigma); DMEM without serum, in the presence of rapamycin (100 nM, LC-Laboratories). Cells were fixed in 4% paraformaldehyde.

### Immunofluorescence

For immunofluorescence analysis of brain sections, right and left hemispheres of dissected brains were separated and rapidly frozen in liquid nitrogen. Sagittal sections (7 μm) were in part collected onto glass slides and in part used for preparing protein extracts, in order to perform comparable analysis. Samples were fixed, permeabilized for 10 min in cold 50% methanol-50% acetone at −20°C and then dried. After washing with a phosphate buffered saline (PBS) solution, samples were incubated for 2.5 hr with 4% BSA IgG-free (Jackson) in PBS, washed in PBS buffer and treated for 30 min with a blocking solution containing 0.05 mg/ml anti-mouse IgG Fab fragment (Jackson Immunoresearch). Collagen VI staining was performed after 1 hr pre-incubation of primary antibody with acetone powder derived from *Col6a1*^−/−^ murine brain tissue, in order to avoid unspecific signals.

For immunofluorescence analysis of *in vitro* primary neural cell cultures, cultures grown on slides were washed in PBS, permeabilized for 3 min at room temperature in PBS containing 0.1% Triton X-100, and incubated for 1 hr with a blocking solution containing 10% goat serum (Sigma) in PBS. Primary antibodies were applied for 2 hr at room temperature or overnight at 4°C, diluted in 5% goat serum in PBS.

The following antibodies were used: rabbit polyclonal antiserum against mouse collagen VI (AS72, kindly supplied by A. Colombatti, Aviano); rabbit and guinea pig polyclonal antisera against mouse α3(VI) (kindly supplied by R. Wagener, Colonia); mouse monoclonal anti-β3-tubulin (Sigma-Aldrich); rabbit polyclonal anti-p62 (Sigma-Aldrich); guinea-pig polyclonal anti-p62 (Progen); mouse monoclonal anti-NeuN (MAB377, Millipore); mouse monoclonal anti-ubiquitin (Cell Signaling). After washing, slides were incubated for 1 hr at room temperature with secondary antibody diluted in 5% goat serum in PBS solution. The following secondary antibodies were used: anti-mouse CY2 (115-226-062, Jackson Immunoresearch); anti-rabbit CY2 (111-225-144, Jackson Immunoresearch); anti-rabbit CY3 (111-165-144, Jackson Immunoresearch); anti-rabbit IRIS5 (5WS-08, Cyanine Technologies); anti-guinea-pig CY2 (706-545-148, Jackson Immuno-research). Nuclei were stained with Hoechst 33258 (Sigma). Slides were mounted in 80% glycerol in PBS and analyzed by fluorescence microscopy.

### TUNEL assay

TUNEL (TdT-mediated dUTP Nick-End Labeling) analysis was performed with the Dead End Fluorometric *in situ* apoptosis detection system (Promega). Brain cryosections were permeabilized for 10 min at −20°C in cold 100% methanol, dried, washed in PBS, treated for 5 min with proteinase K at room temperature, washed again in PBS and finally incubated with equilibration buffer for 10 min. Neural cell cultures grown on slides were first stained for immunofluorescence and then, once washed the secondary antibody, directly incubated with equilibration buffer for 10 min. Samples were then incubated for 1 hr at 37°C with a buffer containing fluorescent nucleotides, rTdT enzyme and Hoechst. SSC solution was used to block the activity of the enzyme, then slides were washed and prepared for microscopy analysis.

### Western blotting

Brain cryosections were lysed in a lysis solution (Tris 50 mM, pH 7.5, NaCl 150 mM, MgCl2 10 mM, DTT 0.5 mM, EDTA 1 mM, glycerol 10%, SDS 2%, Triton X-100 1%) in the presence of phosphatase inhibitors (Cocktail II, Sigma) and protease inhibitors (Complete EDTA free, Roche). Primary neural cells cultured in 6-well plates were washed in PBS and scraped in the same lysis buffer as above. Proteins were quantified with the BCA Protein Assay kit (Pierce). Protein lysates (20 μg) were separated by SDS-PAGE onto 12% or 4-12% polyacrylamide gels (Invitrogen) and blotted onto PDVF membrane (Millipore). Membranes were incubated overnight at 4°C with the primary antibodies, in a 0.1% Tween-TBS (TTBS) solution supplemented with 5% BSA (Sigma). Membranes were then washed three times with TTBS, and incubated for 1 hr at room temperature with HRP-conjugated secondary antibodies (Amersham Bioscience) in TTBS supplemented with 2.5% milk. Detection was performed by chemiluminescence (Pierce). When needed, membranes were stripped using a stripping buffer (25 mM glycine, 1% SDS, pH 2.0) and re-probed. The following primary antibodies were used: rabbit polyclonal anti-LC3 (Sigma); rabbit polyclonal anti-Beclin 1 (Cell Signaling); guinea pig polyclonal anti-p62 (Progen); rabbit polyclonal anti-Bax (N-20, Santa Cruz); mouse monoclonal anti-Bcl-2 (BD Transduction Laboratories); rabbit monoclonal anti-Bcl-X_L_ (clone 54H6, Cell Signaling); mouse monoclonal anti-β-actin (Chemicon). Western blots were performed in at least three independent experiments. Densitometric analysis was carried out using the ImageJ software.

### DHE staining

Brain cryosections were immersed in a PBS solution containing 5 μM DHE (Sigma) and kept at 37 **°**C for 30 min in dark environment. Slides were then washed twice with PBS and prepared for microscopy analysis. Fluorescence intensity was measured by Photoshop software.

### Real time PCR

For RNA extraction, brain cryo-sections were lysed in Trizol reagent (Invitrogen), and processed according to manufacturer instructions. cDNA products were generated with SuperScript III reverse transcriptase (Invitrogen) and analyzed by qRT-PCR with the Rotor Gene SYBR Green PCR kit (Qiagen). Data were normalized to *Gapdh* expression. The following primers were used: *Col6a1* (forward: 5′–TGCCCTGTGGATCTATTCTTCG–3′; reverse: 5′–CTGTCTCTCAGGTTGTCAATG–3′), *Col6a2* (forward: 5′–CTACTCACCCCAGGAGCAGGAA–3′; reverse: 5′–TCAACGTTGACTGGGCGATCGG–3′), *Col6a3* (forward: 5′–AACCCTCCACATACTG CTAATTC–3′; reverse: 5′–TCGTTGTCACTGGCT TCATT–3′), *Gapdh* (forward: 5′–GGGAAGCCCA TCACCATCTT–3′, reverse: 5′–GCCTTCTCCATG GTGGTGAA–3′).

### Behavioural tests

Rotarod performance was measured on a rotating bar using the rotarod device at a fixed speed (30 round per min). Mice were trained for 2 days, in order to get used to the rotating bar, and then tested on three consecutive days. Each day, three runs were conducted with each animal. The last day, the time each mouse stood on the rotating rod was measured and recorded. Ledged beam test was performed on 23-month-old mice using a suspended runway 80 cm long and 6 cm wide at the starting point, which gradually narrowed down to 0.5 cm. The distance from the starting point to the point where the first foot fault occurred was measured, and the number of total hind foot faults and the time spent to cross the runway were recorded. For Y-maze test analysis, mice were tested for spatial memory task by evaluating spontaneous alternation behaviour in a Y-maze. The maze consisted of three arms of the same size. Each arm was a U-shaped corridor of 40 cm length, 5 cm width and 13 cm height, and the arms were oriented at 120° from each other. Each test started by putting the mouse at the end of one arm and allowing it to freely explore the Y-maze in a 8-min session. The sequence of the arm visits was recorded manually and alternation percentage was measured as the number of triads (triplets comprising three different arms) divided by the total visits minus two. An arm choice was defined as both forepaws and hindpaws fully entering the arm [66].

### Statistical analysis

Data are represented as mean ± SEM. Statistical analysis of data was carried out using the Student's t-test. A *P* value < 0.05 was considered as a significant difference.

## SUPPLEMENTARY FIGURES


