# Supplementary material for: Lack of collagen VI promotes neurodegeneration by impairing autophagy and inducing apoptosis during aging
Source: Aging (Albany NY). 2016 Apr 7;8(5):1083–98. doi: 10.18632/aging.100924 (PMC4931855; doi:10.18632/aging.100924)
Supplement: Supplementary file 1 [file aging-08-1083-s001.pdf]

SUPPLEMENTARY FIGURES

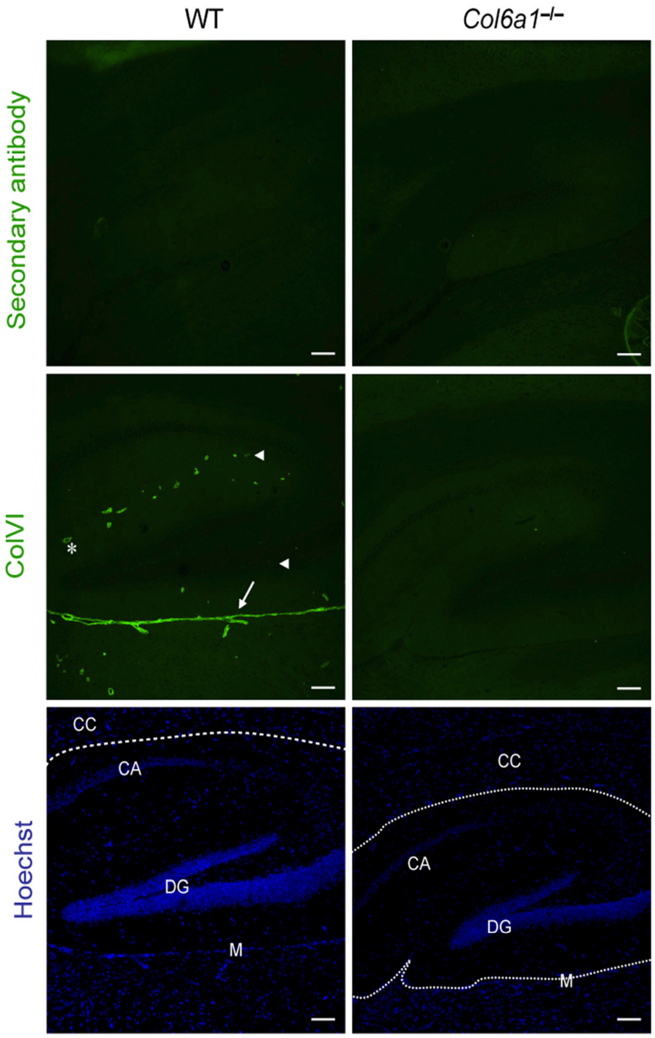

Supplementary Figure 1. Immunofluorescence for collagen VI on brain sections of wild-type and *Col6a1*$^{-/-}$ adult mice. As indicated, a pre-soaked polyclonal antibody against murine collagen VI was used and compared with immunostaining performed in the absence of primary antibody. Labelling for collagen VI is detectable along meninges (arrows) and in small blood vessels (asterisks), as well as inner regions of the hippocampus (arrowheads). Nuclei were stained with Hoechst (blue). Scale bar, 100 μm. CA, Ammon's horn; CC, corpus callosum; DG, dentate gyrus; M, meninges; WT, wild-type.

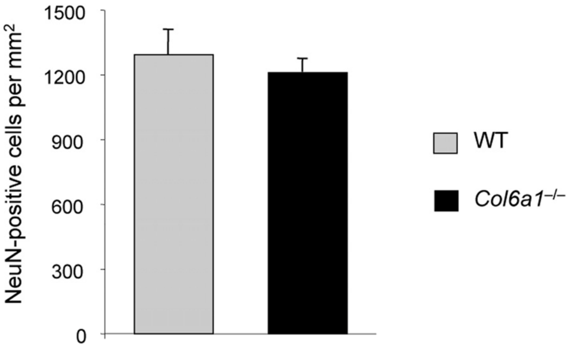

Supplementary Figure 2. Quantification of NeuN-positive nuclei in the cortex of brains of 23-month-old wild-type (n = 3) and *Col6a1*$^{-/-}$ mice (n = 4). The number of NeuN-positive cells was counted and reported per area unit (mm^2). An average of 25 images per animal were analyzed, and no significant difference was revealed from the analysis.

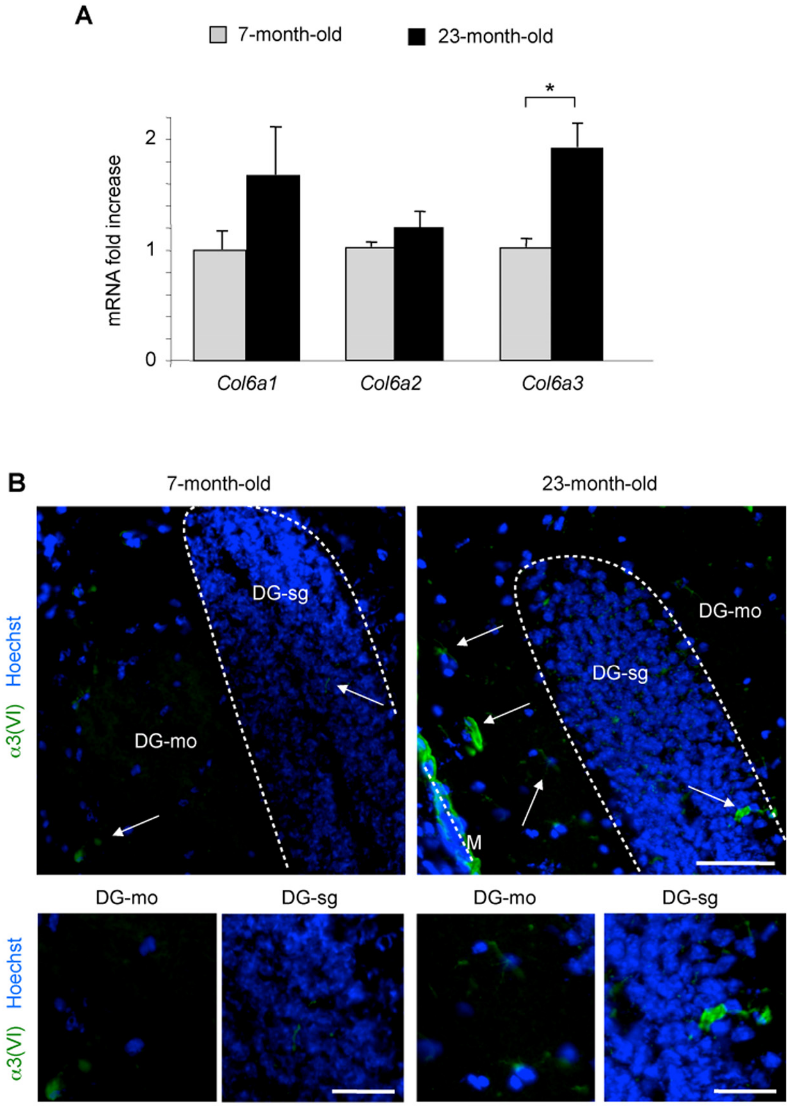

Supplementary Figure 3. (**A**) Quantitative real time RT-PCR analysis of the three collagen VI mRNA transcripts in brain sections of 7-month-old and 23-month-old wild-type mice (*, $P<0.05$; $n = 7$). (**B**) (Upper and lower panels) Immunofluorescence for α3(VI) on brain sections of hippocampal region of 7-month-old and 23-month-old wild-type mice. Two enlargments per picture are displayed in the lower panels. Collagen VI labelling (green) is highlighted by arrows within different cell layers. Nuclei were stained with Hoechst (blue). Upper panels, Scale bar, 50 μm. Lower panels, Scale bar 25 μm. DG-mo, dentate gyrus, molecular layer; DG-sg, dentate gyrus, granule cell layer; M, meninges; WT, wild-type.

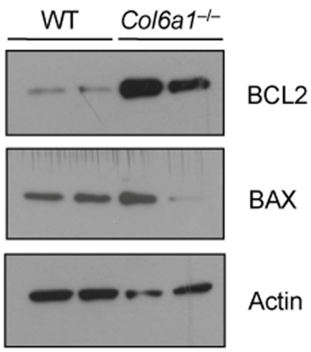

Supplementary Figure 4. Western blot analysis for Bcl-2 and Bax in total protein extracts derived from the brain of 23-month-old wild-type and *Col6a1*$^{-/-}$ mice. Actin was used as a loading control. WT, wild-type.

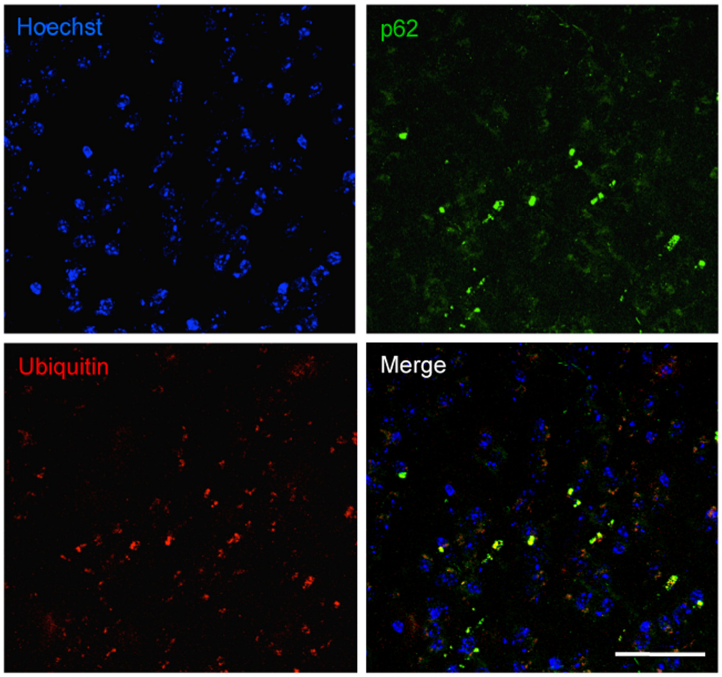

Supplementary Figure 5. Co-immunostaining for p62 (green) and ubiquitin (red) in brain sections of 23-month-old *Col6a1*$^{-/-}$ mice. Nuclei were stained with Hoechst (blue). Scale bar, 50 µm.
